# The Nitrate-Nitrite-Nitric Oxide Pathway: Potential Role in Mitigating Oxidative Stress in Hypertensive Disorders of Pregnancy

**DOI:** 10.3390/nu16101475

**Published:** 2024-05-14

**Authors:** Priscila Oliveira Barbosa, José E. Tanus-Santos, Ricardo de Carvalho Cavalli, Tore Bengtsson, Marcelo F. Montenegro, Valéria Cristina Sandrim

**Affiliations:** 1 Department of Gynecology and Obstetrics, Ribeirao Preto Medical School, University of São Paulo—USP, Ribeirão Preto 14049-900, SP, Brazil; barbosapo@usp.br (P.O.B.);; 2 Department of Pharmacology, Ribeirao Preto Medical School, University of Sao Paulo—USP, Ribeirão Preto 14049-900, SP, Brazil; tanus@fmrp.usp.br; 3 Department of Molecular Biosciences, The Wenner-Gren Institute, Stockholm University, SE-106 91 Stockholm, Sweden; 4 Department of Pharmacology, São Paulo State University—UNESP, Botucatu 18618-689, SP, Brazil

**Keywords:** endothelial cell, pregnancy, nitric oxide, nitrate, oxidative stress, pre-eclampsia

## Abstract

Hypertensive diseases of pregnancy (HDPs) represent a global clinical challenge, affecting 5–10% of women and leading to complications for both maternal well-being and fetal development. At the heart of these complications is endothelial dysfunction, with oxidative stress emerging as a pivotal causative factor. The reduction in nitric oxide (NO) bioavailability is a vital indicator of this dysfunction, culminating in blood pressure dysregulation. In the therapeutic context, although antihypertensive medications are commonly used, they come with inherent concerns related to maternal–fetal safety, and a percentage of women do not respond to these therapies. Therefore, alternative strategies that directly address the pathophysiology of HDPs are required. This article focuses on the potential of the nitrate-nitrite-NO pathway, abundantly present in dark leafy greens and beetroot, as an alternative approach to treating HDPs. The objective of this review is to discuss the prospective antioxidant role of nitrate. We hope our discussion paves the way for using nitrate to improve endothelial dysfunction and control oxidative stress, offering a potential therapy for managing HDPs.

## 1. Introduction

A significant percentage of pregnant women worldwide suffer from hypertension, a common and potentially fatal pregnancy condition. It is estimated that approximately 5% to 10% of all pregnancies are complicated by hypertensive disorders of pregnancy (HDPs), making it one of the leading causes of maternal and fetal morbidity and mortality [1,2]. HDPs are categorized into four main categories: gestational hypertension, pre-eclampsia/eclampsia, chronic hypertension, and chronic hypertension with pre-eclampsia superimposed. Each has distinct characteristics and implications for the health of the mother and fetus [3].

HDPs can harm the placenta and affect the baby’s growth, leading to problems such as low birth weight, restricted growth, and premature birth [4,5]. A malfunctioning placenta is also a major contributor to the development of pre-eclampsia, placing both the mother and the baby at risk [5]. As a consequence, children born from pregnancies complicated by HDPs are more prone to conditions like heart disease and metabolic disorders later in life [6]. For mothers, the repercussions included renal and liver damage, thrombosis, and, in severe cases, cerebral hemorrhage [7].

Although HDPs are a common and potentially serious issue for both mothers and babies, there are limited therapeutic options. Managing conditions related to HDPs typically involves controlling blood pressure and preventing complications [8]. Commonly prescribed antihypertensive drugs include methyldopa, labetalol, and nifedipine [9], and aspirin supplementation may be recommended for specific cases [9]. In the most severe cases, when there is a threat to the life of the mother or the fetus, early delivery may be necessary, even if the baby has not yet reached full gestation maturity.

Managing high blood pressure during pregnancy requires reliable and effective therapeutic intervention. Research into complementary strategies, like dietary interventions, is essential alongside conventional treatments to ensure a safer and healthier pregnancy. Vitamins, minerals, and amino acids have been extensively studied for their potential effects on HDPs [10,11,12,13].

Among this exploration, dietary nitrate intake from natural sources shows promising potential. Many studies center on nitrates’ ability to elevate nitric oxide (NO) levels via the nitrate-nitrite-NO pathway, reducing blood pressure [14,15,16,17,18,19,20]. The nitrate-nitrite-NO pathway is recognized for its influence on vascular relaxation, and reviews published to date usually focus on these vasodilatory properties. However, the antioxidant effects of the nitrate-nitrite-NO pathway in the context of HDPs remain an underexplored avenue deserving further research. Therefore, this review discusses the potential implications of the nitrate-nitrite-NO pathway on HDPs, emphasizing its prospective antioxidant role and underlying mechanisms of the redox system. 

Initially, we will discuss the foundational concepts of HDP pathophysiology: endothelial dysfunction and oxidative stress. From there, we will delve into the core of our discussion—the nitrate-nitrite-NO pathway’s potential as an antioxidant, exploring possible links with the pathophysiology of HDPs. We hope to uncover this pathway’s potential therapeutic value in a context beyond vasodilation, providing a deeper understanding of the mechanisms that might involve the modulation of the redox system.

## 2. Endothelial Dysfunction, Oxidative Stress, and Hypertensive Disorders of Pregnancy

The endothelium, a monolayer of endothelial cells lining the inner surface of blood vessels, regulates fluid and electrolyte flow between the bloodstream and tissues, aids vasodilation, and prevents blood clots in a healthy state [21]. Endothelial dysfunction, characterized by its inability to maintain vascular functions, is a hallmark of HDPs [22,23]. More than being a consequence, emerging evidence suggests that endothelial dysfunction may act as a primary etiology of HDPs [23]. Characteristic manifestations of this dysfunction encompass reduced NO bioavailability, increased inflammatory processes, and enhanced oxidative stress, forming a self-potentiating feedback cycle.

Oxidative stress occurs when there is an imbalance between reactive oxygen species (ROS) production and the body’s capacity to neutralize their harmful effects. Oxidative stress is associated with numerous cardiovascular diseases, including HDPs [24]. During pregnancy, oxidative stress plays a pivotal role in vascular physiological adaptations due to the increasing oxygen and metabolic demands of the growing fetus [25,26]. At physiological levels, ROS are important in cellular signaling and the modulation of vascular function [25] and contribute to the maturation and functionality of the placenta [27]. However, when levels become excessive during pregnancy, that can impair vascular equilibrium and disrupt endothelial harmony, resulting in the endothelial dysfunction characteristic of HDPs.

**Figure 1:**
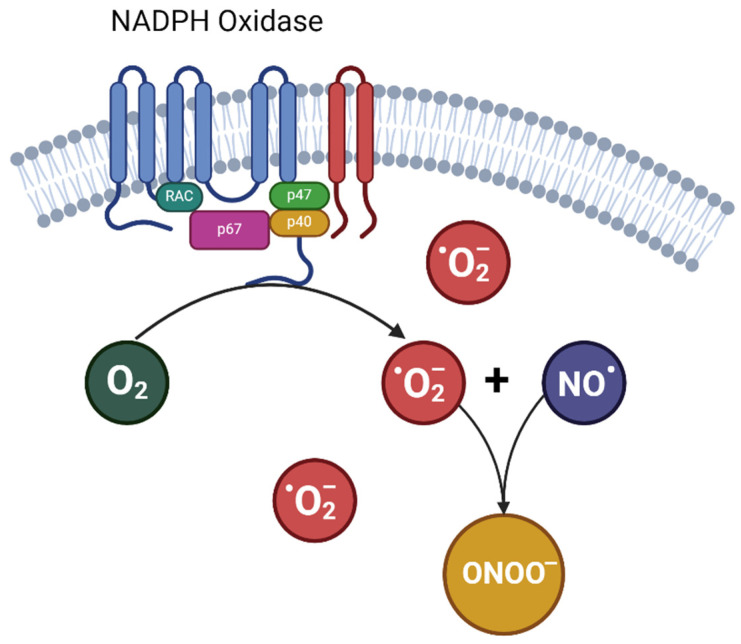
Formation of peroxynitrite from nitric oxide and ^•^O_2_^−^. NO^•^: nitric oxide; ^•^O_2_^−^: superoxide; ONOO^−^: peroxynitrite.

The endothelium’s vulnerability to elevated ROS levels is derived from various metabolic pathways activated in blood vessels during pregnancy, leading to free radical generation. The NADPH oxidase enzyme family is particularly identified as one of the primary sources of superoxide (O_2_^•−^) within the endothelium [28], and there is an upregulation in the expression of NADPH oxidase and its subunits in pre-eclampsia [29,30,31]. As the superoxide production driven by NADPH oxidase increases, this free radical rapidly reacts with vascular NO, forming peroxynitrite (ONOO^−^), an even more toxic radical [32] (Figure 1). More importantly, this fast reaction between superoxide anion and NO reduces NO bioavailability, feeding a detrimental cycle. Indeed, studies assessing peroxynitrite concentrations in pregnant women with hypertensive disorders have shown that elevated levels of peroxynitrite are related to increased vascular damage [33,34].

The phenomenon of the uncoupling of endothelial nitric oxide synthase (eNOS) adds complexity to that scenario [35]. Typically, eNOS converts L-arginine into the vasodilator NO. Under stress conditions, however, its activity is redirected towards producing superoxide anion [36]. This shift is accentuated by the upregulation of NADPH oxidase, leading to excessive superoxide anions. These radicals, known for oxidizing tetrahydrobiopterin (BH_4_)—an indispensable cofactor for eNOS—can contribute to the onset of pre-eclampsia by affecting BH_4_ levels and thereby disrupting eNOS regulation [37]. A study by Chatre et al. (2022) showed the importance of BH_4_ to eNOS function, demonstrating that BH_4_ supplementation might promote eNOS coupling and potentially ameliorate pre-eclampsia symptoms in a mice model [38]. The oxidation of BH_4_ blocks its contribution to NO synthesis and further drives eNOS towards uncoupling, propelling the system further into a pronounced prooxidative state. In a rat model of pregnancy-induced hypertension, the authors observed an increase in superoxide generation and BH_4_ oxidation, indicating the connection between that pathway [39].

Xanthine dehydrogenase (XDH) and xanthine oxidase (XO) are interconvertible forms of an enzyme essential for purine metabolism. Ordinarily, XDH predominates, catalyzing the conversion of hypoxanthine to xanthine and, subsequently, to uric acid, producing NADH in the process [40]. Stressors, such as ischemia, can induce a transition from XDH to XO, which then utilizes molecular oxygen as its electron acceptor, producing superoxide and hydrogen peroxide [41]. Both pre-eclampsia and gestational hypertension have been linked to increased XO activity [42,43].

Lastly, mitochondria, essential to cellular bioenergetics, serve as an established source of ROS, particularly within the placental environment [44]. Acting as the cellular site for aerobic ATP synthesis via oxidative phosphorylation, the importance of mitochondrial function becomes accentuated during pregnancy due to the higher metabolic demands of the placenta [44,45]. The increased mitochondrial activity in the placenta directly correlates with a rise in ROS generation [46]. Inefficient electron transfer can facilitate partial oxygen reduction, leading to an excessive generation of superoxide radicals, as evidenced in HDPs [47,48].

A robust maternal antioxidant defense system regulates metabolic pathways, reducing the harmful effects of oxidative stress and protecting against endothelial dysfunction [49]. However, this appears not to be the case in pregnant women with HDPs. While some studies have reported a decline in maternal antioxidant capacity in HDPs, characterized by a decrease in the activity of enzymes such as catalase (CAT), superoxide dismutase (SOD), and glutathione peroxidase (GPx) [50,51,52], others observed an upregulated antioxidant response [53,54], suggesting a compensatory mechanism to the heightened release of ROS. The indisputable fact is that there is an increase in ROS levels in HDP conditions. Additionally, elevated superoxide anions reduce the bioavailability of NO and have profound implications for endothelium-dependent vasodilation. Beyond these redox system alterations, evidence points to reduced nitrite and nitrate levels in HDPs [55,56]. Contemporary research increasingly postulates that a combination of endothelial dysfunction, oxidative stress, and failure of the NO system coordinate the onset and progression of pregnancy-associated hypertensive disorders.

A key proposition of this review is that the nitrate-nitrite-NO pathway could exhibit antioxidant properties and play a role in modulating oxidative stress. Although this perspective is little explored in the literature, it can have a significant impact on treating HDPs.

## 3. Nitrate-Nitrite-NO Pathway: Antioxidant Dynamics and Therapeutic Implications

**Figure 2:**
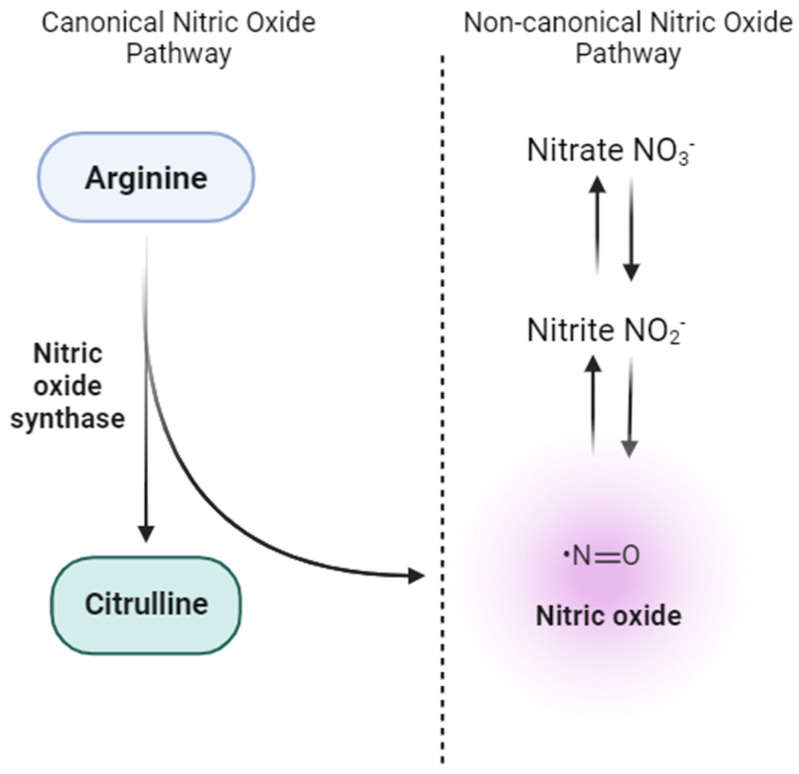
Canonical and non-canonical pathways for nitric oxide (NO) production. The canonical NO pathway involves the enzymatic conversion of L-arginine to NO and citrulline by endothelial nitric oxide synthase (eNOS), predominantly in vascular endothelial cells. On the other hand, the non-canonical NO pathway highlights the stepwise reduction of dietary nitrate (NO_3_^−^) to nitrite (NO_2_^−^) and subsequently to NO in various tissues.

NO is traditionally synthesized enzymatically via the nitric oxide synthase (NOS) pathway. However, research breakthroughs have shown alternative routes of NO generation, most notably by reducing nitrate and nitrite [57,58] (Figure 2). The nitrate-nitrite-NO pathway is increasingly recognized for its critical function in NO dynamic modulation [59]. Because of the volatile nature of NO and the myriad physiological processes that can deplete NO levels in the body, nitrate and nitrite are now perceived as functional reservoirs for stored NO. 

An equilibrium in NO is pivotal for maintaining vascular health, but it becomes vulnerable during detrimental conditions like endothelial dysfunction and oxidative stress. Reviews of clinical trials have underscored the role of nitrate in improving endothelial dysfunction and lowering blood pressure [19,60]. HDPs, such as pre-eclampsia, often exhibit reduced NO levels in the circulatory system, exacerbating vascular complications and elevating blood pressure [61,62]. A deficit in NO has been associated with endothelial dysfunction and overall oxidative stress. As a result, therapeutic interventions based on dietary modifications or supplements aimed at restoring NO balance could hold promise in managing HDPs by addressing endothelial dysfunction.

In exploring the nitrate-nitrite-NO pathway, it is essential to recognize the importance of nitrite, even though dietary sources often contain higher concentrations of nitrate. Following consumption, a substantial portion of the ingested nitrate is reduced to nitrite within the body [59]. Therefore, nitrite acts as an essential intermediate in the body’s nitrate metabolic pathway. To comprehensively discuss the nitrate-nitrite-NO pathway’s antioxidant properties, we must adopt a viewpoint encompassing nitrite-centric findings, understanding the synergistic relationship between nitrate and nitrite in the antioxidant mechanism.

In 2011, Montenegro and colleagues conducted the first study that showed a clear antioxidant effect from the treatment with sodium nitrite treatment in hypertensive animals [63]. By that time, the emerging role of the novel nitrate-nitrite-NO pathway was taking shape, and the first evidence that this newer path could be used to control human blood pressure was shown in 2008 by Andrew Webb and his collaborators [18]. In an elegant study, they demonstrated the influence of organic nitrate from a dietary source (beetroot juice) on human blood pressure and the role of the bioconversion of nitrate to nitrite [18]. However, only healthy volunteers were included. Montenegro’s study questioned the role of the newer pathway in disease scenarios such as hypertension, where the increased oxidative stress could reduce NO bioavailability. They used an experimental model of hypertension 2K1C known for its high activation of the renin-angiotensin system that leads to overexpression of NADPH oxidase. Surprisingly, in addition to the antihypertensive effects induced by nitrite, confirming the potential of the newer pathway even during high oxidative stress conditions induced by hypertension, nitrite treatment dramatically reduced oxidative stress, including in the sham-treated animals [63]. Further investigation depicted a role of nitrite inhibiting the vascular NADPH oxidase, and these experimental findings have been confirmed in several subsequent studies [64,65,66]. 

Experimental studies offer insights into the potential mechanisms of nitrate and nitrite in controlling oxidative stress [66,67,68]. Nitrate has been shown to reduce ROS production, decreasing oxidative markers tied to lipid oxidation, such as TBARS adducts and 4-hydroxynonenal (4-HNE) [69,70,71]. Moreover, short-term nitrite therapy has shown promise in reversing age-related vascular endothelial dysfunction and reducing oxidative stress. This is evident from the restoration of endothelium-dependent dilation and significant mitigation of arterial superoxide production and inflammation in aged male C57BL6J mice [72].

Beyond the effects on lipid oxidation, nitrate and nitrite have exhibited protective mechanisms against protein oxidations—a crucial aspect of oxidative stress. Such modifications can lead to irreversible changes to protein side chains, often compromising cellular function by affecting protein stability and enzymatic activity [73]. In studies spanning both humans and animals, nitrate and nitrite supplementation has demonstrated a dual benefit: it not only ameliorated endothelial function but also resulted in a notable decrease in nitrotyrosine levels—a marker of protein oxidation [74,75,76]. 

In this sense, the potential therapeutic benefits of nitrate for HDPs appear particularly promising. The ability of nitrate-nitrite-NO to mitigate oxidative stress markers suggests its potential in reducing such markers and alleviating endothelial dysfunction, placing nitrate supplementation as a viable path avenue for HDP management.

Elevated markers of lipid and protein oxidation in pregnant women with pre-eclampsia and gestational hypertension are central contributors to the endothelial dysfunction characteristic of HDPs. Studies carry out in women with pre-eclampsia show a pronounced elevation of lipid peroxidation markers [77,78,79,80,81]. Similarly, plasma from women with HDPs consistently reveals heightened levels of protein oxidation, contrasting with samples from women experiencing normotensive pregnancies [82]. Although DNA oxidation markers are less frequently examined, they are also altered in women with HDPs [83,84]. 

The exact mechanism by which nitrate-nitrite-NO exerts these protective roles remains unclear. Nonetheless, based on existing studies, there is growing speculation that these compounds regulate the production of the superoxide radical, targeting its primary sources, namely NADPH oxidase, XOR, and mitochondria. Additionally, considering nitrate and nitrite as reservoirs for NO, it is essential to emphasize their potential to enhance NO levels or their bioavailability. 

Research across various animal models has consistently shown that the potential mechanism behind nitrate’s action and its intermediary nitrite might reside in the inhibition of NADPH oxidase activity [63,66,67]. Although direct neutralization of free radicals by these compounds has been suggested, the potential therapeutic role of the nitrate-nitrite-NO pathway seems to be derived from blocking NADPH oxidase activity and the synthesis of O_2_^•−^ [63,64]. These results align with in vitro studies; in macrophages activated with LPS, the NO derived from nitrite reduced the NOX-dependent superoxide production, further emphasizing the role of nitrite-NO in this mechanism [85]. Such results become especially relevant considering the elevated NADPH oxidase activity observed in HDP contexts [86].

Hypotheses about the underlying mechanisms by which the nitrate-nitrite-NO pathway reduces NADPH oxidase activity and superoxide production have gained prominence in the scientific community. A central hypothesis delves into the role of NO in facilitating protein modifications via S-nitrosylation. S-nitrosylation represents a post-translational protein modification in which a NO group is covalently attached to a cysteine residue in a protein, and this process plays a pivotal role in how NO regulates various proteins [87]. Intriguingly, S-nitrosylation appears to be a crucial mechanism by which NO might govern NADPH oxidase activity. In a cellular model, Qian et al. (2012) uncovered that NO could facilitate the reversible post-translational modification of Nox5, leading to reduced ROS production [88]. 

Beyond the influence of nitrate and nitrite on NADPH oxidase, we cannot dismiss the potential mechanisms for controlling oxidative stress via XOR. Amaral et al. (2015) utilized the deoxycorticosterone-salt (DOCA-salt) hypertensive model and found that sodium nitrite supplementation displayed antioxidant properties [64]. In a separate study centered on the activated macrophage, nitrite supplementation appeared to suppress NADPH oxidase activity, although not through the previously hypothesized S-nitrosation mechanism [89]. The authors suggest that this result might be attributed to changes in Nox2 and XOR [89]. The decrease in XOR activity emphasizes the evolving understanding of the effects of the nitrate-nitrite-NO pathway.

The results of nitrate as an antioxidant are also promising regarding mitochondria control. A study involving rats assessed dietary nitrate supplementation’s ability to mitigate oxidative stress in a hypoxia model. In this research, nitrate provided protection against the reduction in complex I activity and alleviated oxidative stress, thereby boosting NO bioavailability within the myocardium [90].

It is interesting to note that even though nitrate is bioactivated to nitrite, which exerts antioxidant effects, both nitrite and nitrate may also regulate eNOS activity and affect endogenous NO formation [91]. Nitrate supplementation dose-dependently reduced vascular eNOS Ser1177 phosphorylation and increased Thr495 phosphorylation, thus decreasing global eNOS activity, with corresponding attenuation of vascular responses [91]. Similar alterations were observed when nitrite was directly added to vascular preparations, thus indicating a direct inhibitory effect of nitrite on endogenous NO formation [91]. In contrast, there is evidence that nitrite may directly promote eNOS-derived NO formation in hypertensive disorders. Nitrite administration improved endothelium-dependent relaxation in spontaneously hypertensive rats by mechanisms involving eNOS activation to produce NO [92]. These studies lead to contrasting conclusions. However, it is possible that both nitrate and nitrite may exert opposite vascular effects when we compare healthy [91] and hypertensive [92] vessels, with significant differences especially with respect to redox conditions, so that nitrite may exert beneficial effects in disease conditions. Adding more complexity to nitrite/nitrate biology, it is also possible that nitrate may inhibit some of the responses found with nitrite, as previously shown [93]. Both nitrate and nitrite are bioactivated by XOR, and they compete for the catalytic site of the enzyme, and these effects may indeed translate a protection by nitrate against excess nitrite bioactivation to NO by XOR [93]. 

The body’s defense against oxidative stress is complex, and nitrate emerges as a potential contributor to enhancing antioxidant capacities. A study by Cui et al. (2019), utilizing a model of ischemia-reperfusion injury-induced post-skin flap in mice, showed that nitrate restored the enzymatic activity of SOD, GPx, and CAT [94]. Extending these findings to humans, research has explored nitrate’s impact on the enzymatic defense system, especially in the context of exercise, given NO’s vasodilatory role [95]. Another study involving patients with metabolic syndrome noted that acute exercise, combined with the intake of nitrate-rich juice, amplified the expression of MnSOD, GPx, and CAT [96].

In HDP conditions, increased oxidative stress can overload antioxidant enzymes, causing the antioxidant system to operate at or beyond its capacity to neutralize the excessive free radicals, leading to an apparent decline in enzyme activity. Women diagnosed with pre-eclampsia typically display lowered plasma concentrations of enzymes like GPx and SOD, a fact that is observed at the mRNA level for these enzymes [97]. Furthermore, CAT activity appears to be suboptimal in women with gestational hypertension than in those with normotensive pregnancies [98]. Building on this, a study by Qiu et al. (2021) highlighted reduced activities of CAT, SOD, and GPx in preeclamptic placental tissues compared to their regular counterparts [99]. While many studies report a decline in antioxidant activity, findings indicate increased enzyme activity as the body attempts to restore normal homeostasis. That idea is supported by the work of Caldeiras-Dias et al. (2021), where women with pre-eclampsia demonstrated heightened activity in the antioxidant response element (ARE) compared to normal pregnancies [100]. Intriguingly, resveratrol, a well-known antioxidant, enhanced ARE activity [100]. This underscores that, regardless of whether there is a decline or increase in activity, adding compounds with antioxidant potential can bolster antioxidant capabilities.

Delving into the molecular complexity of the antioxidant defense, the Nrf2 pathway needs attention. The ARE region is a direct downstream target of Nrf2 and encompasses a diverse array of enzymes essential for antioxidant and detoxification processes [101]. Nrf2 is pivotal for protecting against oxidative stress, orchestrating the coordinated expression of an array of antioxidant genes [101]. Under basal conditions, Nrf2 is anchored in the cytoplasm, bound closely to the Kelch like ECH-associated protein 1 (Keap1) [101]. In oxidative stress, Nrf2 detaches from Keap1, allowing it to translocate to the nucleus, initiating its protective transcriptional program. Given the conceptual role of Nrf2, one might expect its pathway to be increased in conditions like HDPs. However, studies indicate that the oxidative stress signaling pathway, mediated by Nrf2-Keap1, is compromised in the placentas of women with pre-eclampsia [102,103,104]. Studies in animals have shown the potential of nitrites to influence Nrf2 [75,105]. Fascinatingly, Amaral et al. (2019), utilizing a two-kidney one-clip hypertensive rat model, highlighted nitrite’s capability—as an NO donor—to modulate the transcription factor Nrf2 [105]. Mechanistic investigations suggest that NO might enact its effect-inducing modifications in Keap1, subsequently releasing Nrf2 or through the NO-cGMP pathway, bolstering the notion of NO’s central role in orchestrating the antioxidant response.

In light of evidence highlighting the antioxidant abilities of the nitrate-nitrite-NO pathway, as well as the potential influence on transcription factors like Nrf2, it seems pertinent to explore the therapeutic potential of these compounds in the context of HDPs. The idea of supplementing with nitrate or nitrite to enhance antioxidant defenses in HDPs warrants thorough investigation. Such an approach might not only mitigate oxidative stress but could also offer a new path in the management of HDP by addressing the core oxidative perturbations intrinsic to these conditions. Achieving control over oxidative stress offers a dual advantage: (1) augments the bioavailability of NO and (2) prevents the combination of superoxide with NO, which would lead to the production of the potent oxidant peroxynitrite. In the HDP context, enhancing vasodilation through NO, especially when considering nitrate and nitrite supplementation, is pivotal for blood pressure control and improving endothelial dysfunction. This idea is supported by an intriguing study conducted on a pregnant eNOS^−/−^ mice model, where supplementation with beetroot juice—a known rich nitrate source—reduced blood pressure and improved endothelial function [106]. This outcome is in line with the inhibitory role of nitrite on NADPH oxidase, which reduces oxidative stress, thus increasing NO availability.

Drawing upon dietary sources for nitrate consumption, rather than synthetic derivatives, presents both safety and bioavailability benefits. Foods naturally rich in nitrates, like beetroot and green vegetables, may confer a synergistic health advantage, given the multitude of nutritive constituents they encompass. Although the literature has explored the antihypertensive facets of nitrate-rich foods, especially in the HDP context [107,108], there remains a notable gap in in-depth research on the antioxidant potential of dietary nitrate in this specific cohort.

Endothelial dysfunction and oxidative stress are unequivocal hallmarks of HDPs. However, most human studies concerning nitrate or dietary sources thereof are predominantly concerned with their capacity to modulate blood pressure. We do not intend to minimize the importance of blood pressure control in HDP conditions. Instead, we want to discuss its role beyond the vasodilator action. The outcomes of animal studies addressed in this review reinforce the antioxidant potential of the nitrate-nitrite-NO pathway, as summarized in Table 1.

A point of debate that needs to be highlighted here is the ideal level of nitrate intake to obtain the expected results. Although there is no consensus regarding the necessary amount, most studies in humans have employed amounts ranging from 400 to 2400 mg. These intakes exceed the average nitrate intake, estimated to be between 0.4 and 2.6 mg/kg [109,110]. Such daily intake levels can be easily achieved by consuming dark green vegetables and beetroot. However, supplementation often becomes necessary to attain higher nitrate levels. Innovations in the industry have addressed this need by increasing nitrate concentration and obtaining feasible results through different food preparations using beetroot [111]. For example, concentrated beetroot juice (containing 400 mg of nitrate in 70 mL) is commercially available and has been used in studies on pregnant women without adverse effects [107,112].

Another topic warranting brief exploration is the association of nitrate and nitrite with the formation of nitrosamines—compounds linked to cancer [113]. Often, nitrate and nitrite are utilized as preservatives in processed foods, including sausages, ham, and specific meat types [114]. Consuming high quantities of meat and processed foods has been associated with increased cancer risk [115]. On the other hand, the Mediterranean diet, lauded for its numerous health benefits, is rich in nitrates sourced from leafy green vegetables and beetroot [116]. Beyond nitrate content, these dietary patterns also have high amounts of antioxidants, fiber, and other beneficial compounds that contribute to a balanced redox environment. 

We believe it is necessary to consider the intrinsic nature of nitrate versus its source and intake conditions. The nitrate debate calls for caution, considering the importance of dietary and physiological context. An illustrative example comes from a clinical study on using isolated compounds: while antioxidants like vitamin C and E might be beneficial when consumed through natural sources like fruits—due to their interactions with other compounds—their isolated forms did not yield positive results in pre-eclampsia patients [117]. This discrepancy underscores the complexity of bioactive compounds and their interactions. The mechanisms of action of these antioxidants, including potential synergies with other compounds present in whole foods, might play a pivotal role in their beneficial effects. Interestingly, a review addressing the role of nutraceuticals in HDPs highlights how various components present in foods can benefit blood pressure, including improving vascular function, reducing inflammation, and modulating oxidative stress [118]. Therefore, rather than viewing nitrate as a singular antagonist, it is more judicious to analyze the contexts in which it is introduced and the potential synergistic outcomes that may arise.

## 4. Perspectives and Conclusions

In HDPs, the vascular endothelium’s role in ensuring vasodilator, anti-inflammatory, and anti-thrombotic functions becomes severely compromised. A healthy endothelium produces NO in a well-regulated manner. However, this synthesis is hampered in hypertensive conditions. With the increased oxidative stress and endothelial dysfunction that characterize HDPs, the health and well-being of both the mother and fetus are jeopardized. The potential of nitrate, mainly from natural food sources, to reduce oxidative stress, improve endothelial function, and possibly lower blood pressure through the nitrate-nitrite-NO pathway presents a promising avenue for exploration in HDPs.

The potential of the nitrate-nitrite-NO pathway holds considerable promise regarding both safety and bioavailability. Natural nitrate-rich foods, such as beetroot and leafy greens, provide a matrix of nutrients and compounds that may improve dietary nitrate uptake and metabolic conversion to bioactive forms. Aside from the intrinsic safety of dietary consumption, there is the added benefit that certain foods may provide synergistic health benefits due to the presence of other nutritive constituents. While preliminary evidence is promising [107,108], more comprehensive, well-designed clinical trials are required before recommending nitrate-rich diets or supplements as a complementary treatment in HDPs. The physiological alterations during pregnancy may affect the impact of nitrate and nitrite. Dosage, administration, frequency, and the overall nutritional environment are pivotal factors determining both efficacy and safety. Additionally, the long-term effects on fetal development and maternal health post-partum demand extensive research.

Finally, a few caution notes regarding nitrate or nitrite use during pregnancy should be considered. To our knowledge, no information is available concerning the amounts of nitrate and nitrite that may cross the placental barrier and interact with the biology of the fetus. Nitrate from dietary sources leads to a high plasma concentration (90–152 μM) and nitrite is finely controlled and is kept in lower levels [119]. No previous study has simultaneously assessed nitrite and nitrate concentrations in the mother and the newborn. However, it is known that fetal hemoglobin is more easily oxidized to form methemoglobin by nitrite than adult hemoglobin [120]. Methemoglobin is generated when the ferrous component (Fe^2+^) of hemoglobin is oxidized by oxidants, rendering it incapable of binding O_2_ [121]. Moreover, there is evidence that NADH-methemoglobin reductase activity, which reconverts methemoglobin back to hemoglobin, is lower in infants than in adults [122], which could be looked at with caution. Whether maternal nitrate therapy increases newborns’ methemoglobin concentrations remains to be determined, and further studies are needed. 

In conclusion, this review exploits a novel perspective of the nitrate-nitrite NO pathway’s potential as an antioxidant agent in the context of HDPs—a topic previously underexplored. Our work shines a light on future investigations against the context of limited research on the interplay between nitrate supplementation and its interplay with controlling oxidative stress, endothelial dysfunction, and HDPs. 

## Figures and Tables

**Table 1:** Summary of antioxidant effects on animal studies.

Model	Source	Dose	Time	Main Outcomes	References
Oxidative Stress Hypertension	Nitrite Nitrate	10^−5^ mol/L 10^−2^ mol/L	30 min 1 week	↓ NADPH oxidase activity ↓ Hypertension	[66]
Renal Ischemia-Reperfusion	Nitrate	1 mmol/kg/day	2 weeks	↓ O_2_^•−^ levels ↓ IL-12p70, IL-1β, and IL-6	[67]
High-fat diet	Nitrate	1.0 mmol.kg^−1^.d^−1^	7 weeks	↓ NADPH oxidase-derived superoxide and hydrogen peroxide ↓ p67phox protein expression	[68]
High-fat diet	Nitrate	4 mM	8 weeks	↓ Mitochondrial ROS ↓ 4-HNE	[69]
High-fat diet	Nitrate	4 mM	8 weeks	↓ H_2_O_2_ mitochondrial emission ↓ TBARS adducts ↓ N_3_	[70]
Renal and cardiovascular disease	Nitrate	0.14 or 1.4 NaNO_3_ kg ^−1^	11 weeks	↓ Hypertension ↓ Cardiac and renal damage ↓ MDA, 8-OHdG, and iPF2α	[71]
Aging	Nitrite	50 mg/L	3 weeks	↑ eNOS expression ↓ Nitrotyrosine levels ↓ IL-1B, IL-6, IFNg, and TNF-α	[72]
Hypertension	Nitrite	50 mg/L	2 weeks	↓ Blood pressure ↓ ROS levels ↓ Superoxide Anion ↓ Nitrotyrosine ↓ Nox-4 protein	[74]
Age-associated vascular endothelial dysfunction	Nitrite	50 mg/L	8 weeks	↓ Mitochondrial ROS production ↑ Nrf2	[75]
2K1C hypertension	Nitrite	0.5, 5 and 50 mM	4 weeks	↓ Hypertension ↓ MDA, 8-isoprostane ↓ Vascular ROS production ↓ NADPH oxidase activity	[63]
DOCA-salt hypertension	Nitrite	15 mg/kg	4 weeks	↓ Hypertension ↓ MDA, 8-isoprostane ↓ NADPH oxidase activity ↓ XOR activity	[64]
Hypoxia	Nitrate	0.7 mmol^−1^	2 weeks	↑ Complex I activity ↓ Protein Carbonyls ↓Nitroso-compounds	[90]
IR injury Skin Flap	Nitrate	5 mmol/L	7 days before surgery	↓ Histological lesions and protected cells from apoptosis ↑ SOD, GSH-Px, CAT activity ↓MDA ↓ TNF-α, IL-6	[94]
2K1C hypertension	Nitrite	15 mg/kg	4 weeks	↓ Hypertension ↓ ROS levels ↑ Nrf2 ↑ SOD1, CAT, GPx2, TRDX-1, TRDX-2 mRNA expression ↑SOD and GPx activity	[105]

Legend: 2K1C: 2-kidney, 1-clip; 4-HNE: 4-hydroxynonenal; 8-OHdG: 8-hydroxy-2′-deoxyguanosine; CAT: catalase; DOCA-salt: deoxycorticosterone-salt; GPx: glutathione; GPx2: glutathione peroxidase 2; IFNg: interferon-gamma; IL-12p70: interleukin 12p70; IL-1β: interleukin 1 beta; IL-6: interleukin-6; iPF2α: isoprostane; IR: ischemia-reperfusion; MDA: malondialdehyde; N_3_: 3-nitrotyrosine; ROS: reactive oxygen species; SOD: superoxide dismutase; TBARS: thiobarbituric acid-reactive substance; TNF-α: tumor necrosis factor-alpha; TRDX-1: thioredoxin-1; TRDX-2: thioredoxin-2; XOR: xanthine oxidoreductase. “↓”= reduces “↑”= increases.
